# Supplementary material for: Multi‐dataset identification of innovative feature genes and molecular mechanisms in keratoconus
Source: J Cell Mol Med. 2024 Sep 19;28(18):e70079. doi: 10.1111/jcmm.70079 (PMC11412914; doi:10.1111/jcmm.70079)
Supplement: Supplementary file 2 — Material S2. [file JCMM-28-e70079-s002.zip › 7.GSEA analysis/1.ARL11/KEGG_RIG_I_LIKE_RECEPTOR_SIGNALING_PATHWAY.html]

Details for gene set KEGG\_RIG\_I\_LIKE\_RECEPTOR\_SIGNALING\_PATHWAY[GSEA]

|  || Dataset | ARL11.group.cls#h\_versus\_l |
| Phenotype | group.cls#h\_versus\_l |
| Upregulated in class | l |
| GeneSet | KEGG\_RIG\_I\_LIKE\_RECEPTOR\_SIGNALING\_PATHWAY |
| Enrichment Score (ES) | -0.29898497 |
| Normalized Enrichment Score (NES) | -1.1051804 |
| Nominal p-value | 0.32723576 |
| FDR q-value | 1.0 |
| FWER p-Value | 0.997 |
Table: GSEA Results Summary

  

Fig 1: Enrichment plot: KEGG\_RIG\_I\_LIKE\_RECEPTOR\_SIGNALING\_PATHWAY      
 Profile of the Running ES Score & Positions of GeneSet Members on the Rank Ordered List

  

| SYMBOL | TITLE | RANK IN GENE LIST | RANK METRIC SCORE | RUNNING ES | CORE ENRICHMENT || 1 | IFNA6 | na | 3473 | 0.220 | -0.0649 | No |
| 2 | IFNA14 | na | 4269 | 0.191 | -0.0573 | No |
| 3 | IFNA16 | na | 4914 | 0.169 | -0.0488 | No |
| 4 | TRIM25 | na | 5087 | 0.163 | -0.0277 | No |
| 5 | CASP8 | na | 6150 | 0.133 | -0.0370 | No |
| 6 | IFNA4 | na | 6226 | 0.131 | -0.0182 | No |
| 7 | DDX3X | na | 6855 | 0.115 | -0.0179 | No |
| 8 | TRAF3 | na | 6905 | 0.114 | -0.0010 | No |
| 9 | IFNE | na | 7843 | 0.096 | -0.0128 | No |
| 10 | NLRX1 | na | 8592 | 0.081 | -0.0213 | No |
| 11 | RIPK1 | na | 8643 | 0.080 | -0.0099 | No |
| 12 | IKBKB | na | 8698 | 0.079 | 0.0012 | No |
| 13 | SIKE1 | na | 8896 | 0.076 | 0.0078 | No |
| 14 | MAVS | na | 10073 | 0.059 | -0.0166 | No |
| 15 | DHX58 | na | 10177 | 0.058 | -0.0103 | No |
| 16 | FADD | na | 10496 | 0.054 | -0.0108 | No |
| 17 | CYLD | na | 10631 | 0.052 | -0.0064 | No |
| 18 | MAPK9 | na | 11167 | 0.046 | -0.0145 | No |
| 19 | MAPK11 | na | 11276 | 0.044 | -0.0105 | No |
| 20 | CASP10 | na | 11277 | 0.044 | -0.0034 | No |
| 21 | TRAF2 | na | 11495 | 0.042 | -0.0030 | No |
| 22 | OTUD5 | na | 11917 | 0.037 | -0.0092 | No |
| 23 | MAP3K1 | na | 12394 | 0.032 | -0.0178 | No |
| 24 | TRADD | na | 13011 | 0.027 | -0.0312 | No |
| 25 | MAPK12 | na | 13078 | 0.026 | -0.0289 | No |
| 26 | IL12A | na | 13401 | 0.024 | -0.0344 | No |
| 27 | IFIH1 | na | 13787 | 0.021 | -0.0422 | No |
| 28 | AZI2 | na | 14145 | 0.018 | -0.0497 | No |
| 29 | IFNA2 | na | 15087 | 0.011 | -0.0752 | No |
| 30 | CHUK | na | 15127 | 0.010 | -0.0746 | No |
| 31 | MAPK10 | na | 15299 | 0.009 | -0.0781 | No |
| 32 | PIN1 | na | 15369 | 0.008 | -0.0788 | No |
| 33 | TBK1 | na | 16566 | -0.000 | -0.1132 | No |
| 34 | TBKBP1 | na | 17215 | -0.005 | -0.1312 | No |
| 35 | TANK | na | 17444 | -0.006 | -0.1367 | No |
| 36 | MAPK14 | na | 17567 | -0.007 | -0.1391 | No |
| 37 | IRF7 | na | 18984 | -0.017 | -0.1772 | No |
| 38 | IFNB1 | na | 20512 | -0.028 | -0.2169 | No |
| 39 | MAPK13 | na | 20970 | -0.031 | -0.2251 | No |
| 40 | TRAF6 | na | 21028 | -0.032 | -0.2217 | No |
| 41 | IRF3 | na | 21272 | -0.033 | -0.2233 | No |
| 42 | MAP3K7 | na | 22108 | -0.041 | -0.2409 | No |
| 43 | ATG5 | na | 22396 | -0.044 | -0.2421 | No |
| 44 | TNF | na | 22702 | -0.047 | -0.2433 | No |
| 45 | IKBKG | na | 22799 | -0.048 | -0.2384 | No |
| 46 | ISG15 | na | 23033 | -0.050 | -0.2371 | No |
| 47 | NFKBIB | na | 23780 | -0.060 | -0.2491 | No |
| 48 | NFKBIA | na | 24454 | -0.069 | -0.2575 | No |
| 49 | MAPK8 | na | 24525 | -0.070 | -0.2483 | No |
| 50 | IKBKE | na | 25806 | -0.087 | -0.2713 | No |
| 51 | NFKB1 | na | 26767 | -0.104 | -0.2824 | Yes |
| 52 | IL12B | na | 26869 | -0.106 | -0.2684 | Yes |
| 53 | ATG12 | na | 27016 | -0.109 | -0.2552 | Yes |
| 54 | RNF125 | na | 28179 | -0.136 | -0.2670 | Yes |
| 55 | RELA | na | 29186 | -0.162 | -0.2701 | Yes |
| 56 | CXCL8 | na | 29280 | -0.165 | -0.2463 | Yes |
| 57 | DDX3Y | na | 29353 | -0.167 | -0.2216 | Yes |
| 58 | IFNA8 | na | 31555 | -0.242 | -0.2463 | Yes |
| 59 | IFNW1 | na | 31920 | -0.257 | -0.2157 | Yes |
| 60 | IFNA10 | na | 33230 | -0.339 | -0.1991 | Yes |
| 61 | IFNA7 | na | 33254 | -0.341 | -0.1451 | Yes |
| 62 | CXCL10 | na | 33305 | -0.344 | -0.0915 | Yes |
| 63 | IFNK | na | 33679 | -0.376 | -0.0420 | Yes |
| 64 | IFNA21 | na | 34187 | -0.451 | 0.0156 | Yes |
Table: GSEA details [plain text format]

  

Fig 2: KEGG\_RIG\_I\_LIKE\_RECEPTOR\_SIGNALING\_PATHWAY      
 Blue-Pink O' Gram in the Space of the Analyzed GeneSet

  

Fig 3: KEGG\_RIG\_I\_LIKE\_RECEPTOR\_SIGNALING\_PATHWAY: Random ES distribution      
 Gene set null distribution of ES for **KEGG\_RIG\_I\_LIKE\_RECEPTOR\_SIGNALING\_PATHWAY**
